# The Role of Breast Milk Cell-Free DNA in the Regulation of the Neonatal Immune Response

**DOI:** 10.3390/nu16244373

**Published:** 2024-12-19

**Authors:** Tamim Rezai, Shani Fell-Hakai, Shalini Guleria, Gergely Toldi

**Affiliations:** Liggins Institute, University of Auckland, Auckland 1023, New Zealand; tamim.rezai@auckland.ac.nz (T.R.); sfel730@aucklanduni.ac.nz (S.F.-H.); shaliniguleria14@gmail.com (S.G.)

**Keywords:** cfDNA, mucosal immunity, TLR9

## Abstract

The neonatal period is a critical phase for the development of the intestinal immune system, marked by rapid adaptation to the external environment and unique nutritional demands. Breast milk plays a pivotal role in this transition, yet the mechanisms by which it influences neonatal mucosal immunity remain unclear. This review examines the potential mechanisms by which cell-free DNA (cfDNA) in breast milk may impact neonatal immune development, particularly through Toll-like receptor 9 (TLR9) signalling and gut microbiota interactions. We propose that cfDNA in breast milk interacts with TLR9 on the apical surface of neonatal intestinal epithelial cells, potentially serving as an initial anti-inflammatory stimulus before the establishment of commensal bacteria. This hypothesis is supported by the high concentration and stability of cfDNA in breast milk, as well as the known activation of TLR9 by mitochondrial DNA in breast milk. The review emphasises the need for further empirical research to validate these interactions and their implications for neonatal health, suggesting that understanding these dynamics could lead to improved strategies for neonatal care and disease prevention.

## 1. Introduction

The neonatal period marks a crucial phase in the development of the intestinal immune system, a time characterised by rapid adaptation to the external environment and unique nutritional demands. The transition of the intestinal mucosa from mostly sterile to a microbe-rich environment occurs during childbirth, marking the first contact between microorganisms and the gut epithelium [1]. Breast milk, universally acknowledged as the optimal nourishment for newborns, plays a pivotal role during this transition phase. However, the specific mechanisms by which breast milk influences neonatal mucosal immunity are still unclear [2].

Central to the neonate’s developing innate immune system are pattern-recognition receptors (PRRs), such as Toll-like receptor 9 (TLR9). TLR9 recognises cell-free DNA (cfDNA), pathogen-associated molecular patterns (PAMPs) and damage-associated molecular patterns (DAMPs). Furthermore, intestinal epithelial cells (IECs) uniquely express TLR9 on their surface, which is critical in modulating inflammatory responses within the gastrointestinal (GI) tract through interactions with commensal bacteria [3]. The link between TLR9 activity in IECs and inflammatory pathologies such as necrotising enterocolitis in premature neonates is an area of growing interest [4].

Originally, unmethylated CpG motifs in pathogenic bacterial DNA were thought to be the only ligand for TLR9 proteins [5,6,7]. CpG motifs are regions of DNA where a cytosine nucleotide is followed by a guanine nucleotide in the linear sequence of bases along its 5′ to 3′ direction. In bacterial DNA, these motifs are typically unmethylated, which means they lack a methyl group attached to the cytosine. However, more recent studies suggest a broader interaction spectrum, indicating that TLR9 activation is not strictly sequence-dependent and is influenced by other structural characteristics of DNA [8]. Intriguingly, human mitochondrial DNA (mtDNA), which retains features from its bacterial origins, has been shown to be a TLR9 ligand [9].

This evolving understanding of TLR9′s binding capabilities opens new avenues for exploring how cfDNA in breast milk may impact neonatal immune development. Breast milk, rich in mitochondria and maternal genomic cfDNA, presents a complex milieu of nucleic acids with potential immunomodulatory effects [10,11].

This review examines the potential mechanisms by which cfDNA may influence neonatal immune development, focusing on TLR9 and the impact of the gut microbiota. Our exploration provides a context for a speculative hypothesis that aims to elucidate the relationship between breast milk-derived cfDNA and neonatal immunity. Although this hypothesis is currently theoretical, it seeks to inspire further research and stimulate discussion within this emerging field.

## 2. Newborn Immunity

The neonatal immune system, particularly within the gastrointestinal tract, is distinctly different from that of adults. Neonates primarily rely on their innate immune system, which provides rapid and non-specific protection against pathogens. Unlike adults, whose adaptive immune systems are well-developed and capable of recognising and responding to specific antigens, newborns have an immune system that is more geared towards immediate defence. This reliance on innate immunity involves mechanisms such as the production of IL-8 and the activation of innate immune cells like neutrophils, macrophages, and mast cells, which can respond quickly to infections [12].

Memory T cells, T-helper 1 (Th1), T-helper 17 (Th17), and cytotoxic T cells in neonates significantly differ from those in adults. In neonates, the population of memory T cells is limited due to their lack of previous exposure to pathogens, whereas adults have a robust pool of memory T cells that enable a faster and more effective response upon re-exposure to antigens [13]. Th1 cells, which are essential for cellular immunity and combating intracellular pathogens, and Th17 cells, which play a crucial role in mucosal immunity and inflammation, are also less prevalent and less functionally mature in neonates compared to adults [14]. Similarly, cytotoxic T cells, which are pivotal in directly killing infected cells, are present but less prevalent and exhibit a reduced cytotoxic capacity in neonates [13]. This underdevelopment of the adaptive immune system in neonates highlights their reliance on innate immune mechanisms for initial defence against infections.

Neonates often exhibit abundant regulatory T cells (Tregs) and a skewed T-helper 2 (Th2) response, which can be attributed to the regulatory environment established by their innate immune system [13]. This may help mitigate potential inflammatory damage during early development and is fundamental to their survival, protecting developing tissues by tempering immune reactions. However, it can also make neonates more susceptible to certain infections and allergic conditions and rely on maternal antibodies for passive immunity [15]. This anti-inflammatory stance is not passive; it shapes the maturing immune system, influencing its long-term functionality and the GI tract’s health. Understanding these foundational aspects of neonatal immunity is crucial as we delve into the interactions between the developing immune system, the gut microbiome, and factors such as cfDNA, which play significant roles in early-life immune responses.

## 3. Immune Tolerance

This inherent bias sets the stage for developing immune tolerance and is driven by the neonate’s need to adapt to the external environment. Immune tolerance is the ability to differentiate self from non-self and harmless from harmful, which is essential right from birth when neonates face a sudden influx of bacteria and other immunostimulatory molecules. Establishing symbiosis is crucial for establishing a healthy microbiome and an effective immune response, shaping the neonate’s early development and future immunity while also protecting vulnerable tissues from excessive immunological damage [1]. The neonatal immune system develops simultaneously with the establishment of the microbiome; therefore, immune tolerance is crucial for adaptation and setting the stage for lifelong immunity that can respond appropriately to pathogenic threats. The emerging tolerance aims for appropriate responses to pathogens and harmony with benign elements, a balance pivotal for the neonate’s growth and development [1].

However, mucosal surfaces like the GI tract encounter complex challenges, striking a balance between preserving organ functionality by maintaining tolerance towards benign environmental, nutritional, and microbial antigens and effectively activating immune responses against invasive pathogens. At birth, critical gastrointestinal tract functions such as intestinal motility, barrier function, and mucosal immunity are still in the early stages of development [16]. In their nascent and vulnerable state, these systems must rapidly adapt to various external stimuli, a difficult task considering neonatal digestive tracts can take 3-6 months to mature [1]. Recognising the nuances of neonatal immune development, particularly within the gastrointestinal tract, necessitates innovative research approaches. Often relying on animal models, notably mice, researchers seek to overcome ethical and practical constraints associated with human neonatal studies. While invaluable, these models present challenges due to differences in timing and specifics of intestinal development, immune system maturation, and gut microbiota composition between mice and humans. These differences, especially notable in aspects like TLR9 receptor expression and function, necessitate a cautious approach in applying findings from mouse models to human neonates. Such models remain crucial despite these challenges, provided their limitations are acknowledged and findings are validated through complementary studies.

## 4. Cell-Free DNA in Breast Milk: Novel Insights and Implications

The term “cell-free DNA” refers to DNA molecules not enclosed within cellular structures detected in various biological fluids, including blood, saliva, urine, and breast milk. In healthy individuals, cfDNA predominantly originates from normal cellular turnover, known as self-cfDNA, supplemented by contributions from the microbiome [17,18]. Past research has focused heavily on the use of human cfDNA in prenatal testing, cancer screening and diagnostics. However, its specific impact on neonatal development as a component of breast milk remains exploratory [11,19,20].

Through advances in oncological research, studies have found that breast milk is rich in maternal cfDNA, and the mitochondria that are present in breast milk likely add to this composition of cfDNA [10,11,20]. In contrast to plasma cfDNA, the concentration of total cfDNA in breast milk is minimally affected by systemic dilution and clearance mechanisms. Consequently, cfDNA levels in breast milk are approximately 90 times higher than those in plasma [20]. This plenitude of cfDNA in breast milk introduces intriguing possibilities for its impact on the neonatal IECs that express TLR9 receptors [9]. So far, evidence shows that cfDNA from commensal bacteria interacts with adult and neonatal IECs, dampening inflammatory responses caused by other innate immune cells [1,2,4]. While these interactions are still being investigated, they highlight the potential of cfDNA as a component of breast milk to influence neonatal gastrointestinal physiology immediately after birth, during the initial stages of microbiome colonisation.

cfDNA must retain enough core characteristics over time to influence intestinal mucosa as it traverses the neonatal digestive system. Current research shows that cfDNA in breast milk demonstrates remarkable stability, maintaining its structure and quantity for up to seven days at room temperature—a stark contrast to circulating plasma cfDNA, with a half-life ranging from 15–120 min [11,21]. Sender et al. also noted that the concentration of cfDNA in breast milk samples increased over time, likely released by degraded cells within the milk. The stability of cfDNA from breast milk in the neonatal gastrointestinal tract is still an emerging area of research. However, factors such as the neonatal GI tract’s relatively low acidity and enzyme activity, as well as the binding of cfDNA to proteins and lipids in breast milk, may influence its stability and potential functionality. This characteristic stability supports the idea that cfDNA from breast milk could sufficiently maintain its structure as it travels the neonatal digestive system long enough to influence the developing mucosa. Future research should focus on determining the precise mechanisms that preserve cfDNA stability within the neonatal GI tract and evaluating how these factors may vary among infants. Such studies could provide critical insights into the optimal conditions required for cfDNA to exert its immunomodulatory effects.

The majority of cfDNA is derived from the noncoding regions of the genome, similar to the composition of the human genome. This dominance of noncoding sequences is due to cfDNA originating from fragmented genomic DNA, which itself is predominantly noncoding (e.g., intergenic regions, introns, repetitive sequences). However, the relative proportion of coding versus noncoding sequences in cfDNA may be influenced by additional factors, such as the tissue of origin, the mode of cell death (e.g., apoptosis vs necrosis), and pathological conditions like cancer or infections. For instance, cfDNA derived from rapidly proliferating tumour cells or immune cells undergoing activation may show enrichment in certain coding or regulatory sequences [22,23,24,25,26]. While plasma cfDNA originates from various cells, the genetic composition of cfDNA in breast milk is primarily derived from maternal breast cells and closely mirrors the maternal breast’s genetic profile [27]. This intriguing genetic link is made more interesting by a study aiming to catalogue the specifics of cellular DNA turnover and its contribution to circulating cfDNA levels [21]. Using comparative analysis between observed cfDNA levels from various cell types and predictive values derived from existing literature, the study revealed that breast epithelial cells exhibit a distinct pattern of local DNA utilisation. Despite predictions indicating the presence of trace amounts, breast epithelial cell-derived cfDNA was found to be below detectable limits in circulation, underscoring the complex dynamics of cfDNA production and its potential implications in maternal-infant communication during breastfeeding [21]. The existence of a unique mechanism within breast epithelial cells for managing DNA turnover may be more purposeful than we currently understand and may play a role in targeting maternal-infant signalling through cfDNA in breast milk. However, this concept remains speculative and requires further scientific validation.

The unique properties and high concentration of cfDNA in breast milk, combined with its remarkable stability, imply that it may remain intact and functionally active by the time it reaches the neonatal small intestine, where it might be able to exert immune-modulating effects. Nevertheless, this area remains largely unexplored, calling for focused research to unravel the intricacies of breast milk-derived cfDNA’s interaction with the neonatal immune system. Such understanding could significantly advance our knowledge of neonatal nutrition and immune development, bridging a crucial gap in neonatal care research.

## 5. Interaction of cfDNA with the Immune System and TLR9 Expression in Intestinal Epithelial Cells

The innate immune system serves as the first line of defence against infections and heavily relies on pattern-recognition receptors (PRRs) such as Toll-like receptors (TLRs) to identify and respond to invading pathogens. Among these, TLR9 plays a pivotal role in recognising DNA-derived pathogen-associated molecular patterns (PAMPs) and damage-associated molecular patterns (DAMPs). TLR9, a transmembrane protein, is primarily situated intracellularly in immune cells but has selective expression on the surface of particular cells, such as intestinal epithelial cells (IECs). TLR9 is particularly adept at detecting unmethylated CpG motifs, which are prevalent in bacterial DNA [5,7]. cfDNA contains fragments of genes from both nuclear DNA (nDNA) and mitochondrial DNA (mtDNA), which act as signalling molecules and can interact with the immune system. For example, TP53 and KRAS mutations in nDNA-derived cfDNA fragments might influence anti-tumour immunity and trigger inflammation, respectively. Similarly, mtDNA acts as a DAMP that activates TLR9, contributing to inflammatory responses [5,7,24,25]. 

The composition of cfDNA, whether coding or noncoding, may affect its interaction with the immune system. Noncoding cfDNA sequences, such as repetitive elements and CpG-rich regions, can serve as DAMPs and act as potent activators of TLR9. Additionally, fragments from transposable elements or satellite DNA might also enhance immune responses. On the other hand, coding cfDNA, such as fragments containing mutated or aberrantly expressed genes like TP53 or KRAS, can modulate immune responses indirectly by signalling tumour-derived stress or damage, thus amplifying inflammation and immune surveillance in cases such as cancer or infection [25]. TLR9 expressed by IECs is crucial for immune surveillance within the gut, where it plays a significant role in moderating immune responses to the diverse array of microbial life present in the intestinal lumen [3]. The concentration of cfDNA varies depending on physiological and pathological factors, such as tissue-specific damage or cancer. Healthy individuals typically exhibit lower cfDNA levels, while patients with conditions like cancer or significant tissue injury may present with elevated cfDNA concentrations. These differences in cfDNA levels and heterogeneity can modulate immune responses, potentially influencing inflammation, immune surveillance, and the recognition of DAMPs [24,25].

**Figure 1:**
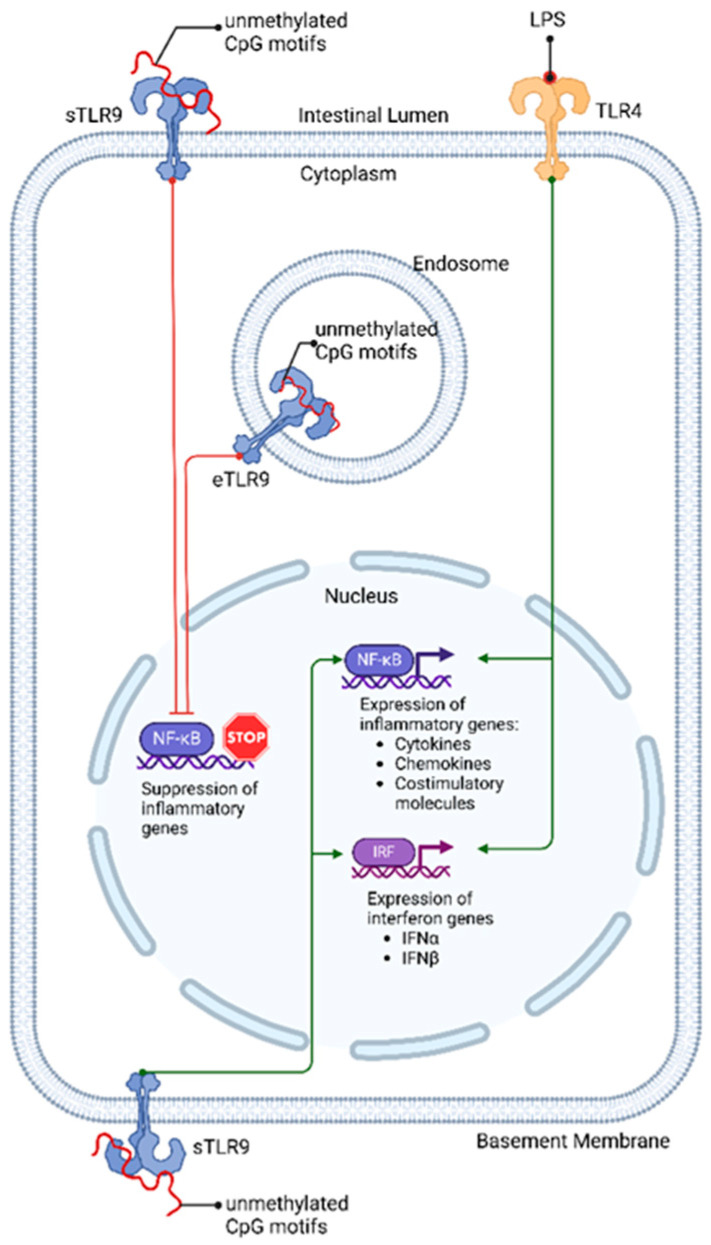
The molecular mechanism of Toll-like receptor 9 (TLR9) signalling pathways in response to unmethylated CpG motifs in intestinal epithelial cells (IECs). Both surface TLR9 (sTLR9) and endosomal TLR9 (eTLR9) bind unmethylated CpG motifs, leading to the suppression of inflammatory gene expression through NF-κB inhibition. In contrast, basolateral TLR9 and apical TLR4 activate NF-κB, driving the expression of inflammatory genes, including cytokines, chemokines, and costimulatory molecules. Additionally, they trigger interferon-regulatory factors (IRF) to promote interferon gene expression, ultimately enhancing inflammation [3].

In its inactive state, intracellular TLR9 resides within the endoplasmic reticulum, moving to endosomes and lysosomes upon activation (Figure 1), where the acidic environment of these compartments is crucial for TLR9 binding and ligand preparation, as optimal TLR9 binding requires a pH range from 6.5 to 5.0 [28]. Activation of TLR9 initiates diverse signalling pathways, such as the interferon regulatory factor (IRF)-mediated antiviral defences and NF-κB pathway-triggered inflammation, resulting in either inflammatory responses that protect the host from invasion or the dampening of inflammatory responses that may cause excessive tissue damage [24,29].

TLR9 proteins are most known for their affinity for bacterial DNA with high concentrations of unmethylated CpG motifs. However, factors beyond a molecule’s DNA sequence can influence TLR9 binding. The curvature and length of the DNA structure, influenced by proteins like high mobility group box 1 (HMGB1) and histones that change how the DNA molecule bends, all strongly influence TLR9 ligation, independent of DNA sequence [8]. Human self-DNA has been shown to activate TLR9 proteins in diseases such as systemic lupus erythematosus (SLE) and atherosclerosis, and mtDNA [8] can cause inflammatory responses through TLR9 interactions [30,31]. Clearly, TLR9 receptors have a wider, more versatile scope than initially believed, suggesting a more complex immune recognition and response system behind TLR9 ligation.

The expression of TLR9 in IECs plays a crucial role in gastrointestinal immune responses. IECs, forming the protective lining of the intestinal lumen, express TLR9 receptors in a uniquely polarised manner on their cell surface. This architectural arrangement of TLR9 receptors on IECs serves a dual purpose: receptors facing the intestinal lumen predominantly interact with commensal bacteria, while those oriented towards the basement membrane are primed to detect potential pathogenic invasions [3]. This strategic polarisation of TLR9 receptors leads to location-dependent differential responses, as stimulation of TLR9 on the basolateral side activates the NF-κB pathway, triggering an inflammatory response, while apical stimulation by commensal bacteria tends to dampen inflammatory responses. This mechanism of apical stimulation, being anti-inflammatory, is thought to counteract inflammation induced by lipopolysaccharide activation of TLR4 receptors that are also expressed apically by IECs [4,32]. TLR4 activation is associated with increased enterocyte apoptosis and reduced mucosal healing [33], while TLR9 activation tends to foster a tolerogenic environment essential for a balanced immune response. This protective role of TLR9 is demonstrated by positive outcomes from experimental treatments using synthetic CpG-based TLR9 agonists on adults and mice with ulcerative colitis, reducing symptoms by targeting the imbalance between mucosal TLR9 and TLR4 activation [34].

This delicate balance orchestrated by TLR9 in IECs is particularly critical in the context of neonatal immunity, as the neonatal gut encounters a myriad of external antigens for the first time. During this period, the gut must learn to distinguish between harmless commensal microbes and potential pathogens. The polarised expression of TLR9 in IECs plays a pivotal role in this process, ensuring a controlled, non-inflammatory state is maintained while still being prepared to mount an appropriate immune response to genuine threats. This intricate mechanism underlines the importance of TLR9 in shaping the neonatal gut’s immune landscape and highlights the need for further research to understand its full implications in neonatal health and disease.

As previously mentioned, studies on intracellular TLR9 receptors highlighted the need for an acidic environment for TLR9 ligation [28]. The mean postprandial pH range in neonatal gastric contents was found to be 5.59 ± 0.22, ideal for TLR9 binding [35,36]. Assuming breast milk-derived cfDNA is stable enough to reach IECs intact, this acidity may indicate that the neonatal gut is an environment where orally introduced cfDNA could effectively interact with apically expressed TLR9 proteins, potentially influencing neonatal immune development.

This evolving understanding of TLR9′s interactions with cfDNA, especially in neonates, underscores the need for more empirical research. Investigating how neonates respond to different types of cfDNA is crucial. Such studies are instrumental in deciphering the unique impact of these molecular entities on the development of early immune responses. Understanding these interactions could offer significant insights into neonatal immunology, potentially leading to novel approaches in neonatal care and immune system modulation. 

## 6. Necrotising Enterocolitis as a Specific Example

Necrotising enterocolitis (NEC) is an inflammatory bowel disease primarily affecting premature infants, who face a higher risk and more severe outcomes compared to full-term infants. It serves as a valuable model for comprehending the anti-inflammatory aspects of TLR9 signalling in neonates, as it involves the dysregulation of these innate immune responses.

Factors like asphyxia, polycythaemia, congenital heart disease, or gastrointestinal anomalies can trigger NEC in full-term neonates, however, in preterm infants, immaturity of the gut and immune system are the primary concern [16]. With their underdeveloped gut microbiota and immune systems, premature neonates may struggle to combat bacterial challenges after birth. The absence of mature gut microbiota, essential for immune tolerance and defence against pathogens, heightens their vulnerability [37]. Very low birth weight (VLBW) neonates experiencing NEC often suffer from severe long-term complications, including short bowel syndrome, malabsorption, neurodevelopmental impairments, and sensory deficits, highlighting the profound impact of NEC on this fragile population. 

In their 2021 study, Shaw et al. identified two distinct pathophysiological mechanisms implicated in NEC among neonates: “CpG-associated NEC” and “Gram-negative-associated NEC.” CpG-associated NEC is characterised by a potential under-stimulation of TLR9 receptors, as evidenced by low levels of bacterial DNA containing CpG motifs in stool samples and decreased bacterial concentration. It is associated with increased postnatal and shorter gestational ages. On the other hand, Gram-negative-associated NEC is characterised by a heightened presence of gram-negative bacteria in the gut, which triggers an overstimulated immune response through TLR4 receptors. This condition often requires longer antibiotic treatment periods and typically occurs in neonates born at a later gestational age and at greater postnatal days of life. These distinct factors underscore the different pathological pathways and outcomes between ‘CpG-associated NEC’ and ‘Gram-negative-associated NEC,’ highlighting the complexity and variability of NEC pathogenesis in neonates. 

Building on the findings of Shaw et al. (2021), further exploration into the role of TLR9 signalling, especially in the context of “CpG-associated NEC,” is imperative. This form of NEC is notably associated with reduced TLR9 activity, potentially attributed to colonisation by bacteria with low CpG frequency, such as C. perfringens and Staphylococcus taxonomic groups. This decrease in CpG concentration may hinder mucosal cells’ ability to counterbalance TLR4 activity effectively through TLR9 regulation. The scarcity of TLR9 activation is concerning because TLR9 signalling in IECs is critical for initiating and regulating immune responses crucial for gut health. It includes maintaining the gut barrier, which is essential for preventing the entry of harmful substances into the bloodstream while facilitating nutrient absorption. Such a microbial imbalance and consequent under-stimulation of TLR9 might contribute to NEC’s inflammation and gut damage.

These disruptions are believed to play a significant role in both the onset and progression of NEC, offering valuable insights into the specific vulnerabilities of VLBW neonates and the mechanisms driving NEC.

## 7. Future Directions

Based on the above insights, several promising avenues for future research are evident. These directions aim to deepen our understanding of the role of cfDNA in breast milk and its interaction with the neonatal immune system, particularly focusing on TLR9 signalling and neonatal intestinal development.

To begin with, in vivo studies are crucial for establishing the stability and functionality of cfDNA in breast milk as it traverses the neonatal digestive system. Conducting these studies in animal models will explore that the structural integrity of cfDNA is preserved and its immunomodulatory potential is maintained up to the point of interaction with neonatal IECs [11,21].

In addition, detailed mechanistic studies are necessary to elucidate how cfDNA from breast milk activates TLR9 receptors on the apical surface of neonatal IECs [9]. These studies should focus on the structural characteristics of cfDNA that facilitate TLR9 binding, such as DNA sequence, length, curvature, and methylation status [8]. Understanding these nuances will provide deeper insights into the specific interactions at play, enhancing our comprehension of neonatal immune modulation. While TLR9 expression has been studied in adult humans, its expression in human neonates remains unexplored [38]. Investigating TLR9 expression in neonates represents a crucial step toward understanding how this receptor functions during the early stages of immune development.

An intriguing area for research lies in the unique mechanism within breast epithelial cells for managing DNA turnover. The genetic composition of cfDNA in breast milk, primarily derived from maternal breast cells and mirroring the maternal breast’s genetic profile, suggests a specialised role for these cells in processing and packaging cfDNA for secretion into breast milk. Investigating this mechanism could reveal novel pathways and regulatory processes essential for maternal-infant communication and immune system priming in neonates. Such research may uncover how breast epithelial cells selectively package cfDNA and the signals that regulate this process, providing a deeper understanding of its role in neonatal development and immune modulation [21,27].

Comparative analyses of the effects of cfDNA and bacterial DNA on neonatal IECs and TLR9 signalling will also be essential [5,7,9]. Such studies will clarify the relative contributions of maternal and microbial factors in early immune development, exploring whether cfDNA can mimic the anti-inflammatory effects of commensal bacterial DNA in neonates [3,4,32]. This comparative approach will help distinguish the unique roles and interplay between different sources of DNA in shaping neonatal immunity.

Future research should investigate how cfDNA in breast milk influences the colonisation and composition of the neonatal gut microbiome. This includes studying whether cfDNA can affect microbial diversity, the establishment of beneficial microbial communities, and the suppression of pathogenic bacteria. Longitudinal studies monitoring the gut microbiome’s development in neonates exposed to breast milk cfDNA could provide valuable insights into how maternal cfDNA shapes early-life microbial ecosystems and their impact on immune responses [4,39,40].

Moreover, longitudinal studies tracking the development of the neonatal immune system in relation to cfDNA exposure from breast milk will help identify critical windows during which cfDNA has the most significant impact. Monitoring immune markers, TLR9 expression levels, and the establishment of immune tolerance over time will provide a temporal dimension to the effects of cfDNA, highlighting periods of heightened sensitivity and influence. Studies have shown that maternal factors, including breast milk composition, significantly influence neonatal immune development [41,42], and the transfer of maternal immune components via breastfeeding plays a crucial role in shaping the infant’s immune responses [43].

Interventional studies using synthetic cfDNA analogues designed to activate TLR9 can test the hypothesis under controlled conditions [34]. By determining the therapeutic potential of cfDNA in preventing or mitigating inflammatory conditions in neonates, such as NEC, researchers can translate theoretical concepts into clinical applications [44]. These studies will pave the way for innovative treatments and preventive strategies.

While TLR9 is the focus of this hypothesis, it is essential to investigate other pattern-recognition receptors (PRRs) and their potential interactions with cfDNA [4,9,32]. Expanding the scope to include other PRRs will provide a more comprehensive understanding of how breast milk influences neonatal immune development through multiple signalling pathways [44]. This broader perspective will enrich the overall framework of neonatal immunology.

By pursuing these directions, we can significantly advance our knowledge of the complex interactions between cfDNA in breast milk and the neonatal immune system. This will ultimately contribute to improved strategies for promoting neonatal health and preventing inflammatory diseases in this vulnerable population.

## 8. Conclusions

In this review, we have explored the intersection of neonatal immune development and the influence of breast milk-derived cfDNA, focusing on TLR9 interactions. The moderated immune response of neonates, crucial for their survival and development, might be significantly shaped by cfDNA interactions, suggesting a maternal influence extending beyond mere nutrition.

Our current understanding suggests that cfDNA in breast milk may play a crucial role in modulating neonatal immune responses by interacting with TLR9 expressed on the apical surface of neonatal IECs. This interaction acts as an initial anti-inflammatory stimulus before the establishment of commensal bacteria. It introduces the concept of breast milk cfDNA as a potential key influencer in the early stages of neonatal gastrointestinal and immune development, suggesting that this mechanism may play a vital role in developing immune tolerance within the neonatal GI tract. Recent advancements in understanding TLR9 indicate its capacity to bind to cfDNA regardless of CpG motifs, although it demonstrates a higher affinity for unmethylated CpG sequences [3,8]. This broader binding capability, influenced by factors such as molecule size and shape, supports the hypothesis that cfDNA from breast milk could interact with TLR9 receptors despite being mammalian DNA. These interactions occurring on the apical surfaces of IECs may potentially serve as an initial anti-inflammatory stimulus in neonates; given the high concentration of cfDNA in breast milk and its notable stability, it is highly likely that maternal cfDNA can traverse the neonatal digestive system and bind to TLR9 receptors on IECs [11,20].

The upregulation of mitochondria, and consequently mtDNA, during lactation further supports our hypothesis [45], as mitochondrial DNA is known to activate TLR9 proteins with a structure that parallels their bacterial origins [30]. This connection provides a biochemical rationale for the hypothesised interaction between breast milk cfDNA and neonatal TLR9 receptors, as mtDNA is a confirmed ligand of TLR9. Furthermore, considering the development of the neonatal GI system, particularly in preterm infants, this hypothesis raises the possibility that underdeveloped intestinal cells in these infants may express less apical TLR9, leading to reduced inflammation dampening [12].

While the role of TLR9 in recognising bacterial DNA is well-documented, recent evidence suggests it may also interact with various cfDNA types, including those from breast milk. This broader functionality implies a potentially complex role in shaping the neonatal gut immune environment. Additionally, the interactions between gut microbiota and TLR9 emphasise the need for more targeted research to understand these intricate dynamics fully. Despite the promising potential of this field, it is essential to recognise the speculative nature of some aspects. Though rooted in current understanding, the hypotheses presented here need empirical validation. Future research should aim to clarify the characteristics of breast milk cfDNA, its exact interactions with neonatal TLR9, and the implications for neonatal health and disease prevention.

Ultimately, understanding the nuanced ways maternal factors like breastmilk cfDNA influence neonatal immune development promises to improve neonatal care and outcomes. As we refine our understanding, we move closer to translating this knowledge into practical strategies for early immune support and disease prevention in neonates, as well as improved long-term immune health outcomes and a reduced burden of allergies and autoimmune disorders in later life.
